# Personality traits and quality of life among Lebanese medical students: any mediating effect of emotional intelligence? A path analysis approach

**DOI:** 10.1186/s40359-022-00739-2

**Published:** 2022-02-11

**Authors:** Elise Maalouf, Souheil Hallit, Sahar Obeid

**Affiliations:** 1 Department of Life and Science, University of Paris-Est, Paris, France; 2 Faculty of Medicine and Medical Sciences, Holy Spirit University of Kaslik (USEK), Jounieh, Lebanon; 3 Research Department, Psychiatric Hospital of the Cross, Jal Eddib, Lebanon; 4 Social and Education Sciences Department, School of Arts and Sciences, Lebanese American University, Jbeil, Lebanon

**Keywords:** Personality traits, Emotional intelligence, Quality of life, Medical students

## Abstract

**Background:**

Medicine is an incredibly demanding career that appears to leave many medical students at risk of fatigue, anxiety, depression and burnout. Since adaptation and lifestyle changes are important, quality of life (QOL) of students during medical school could be impaired. Personality traits and emotional intelligence (EI) facets may be both linked to medical student’s QOL. To our knowledge, no studies have been done on the concurrent and prospective relationship between QOL-related personality traits and EI in Lebanese medical students. This study aimed to investigate the role of EI as a mediator between personality traits and QOL among a sample of Lebanese medical students.

**Methods:**

This research is a descriptive cross-sectional survey study involving 293 Lebanese medical students recruited from all 7 Faculties of Medicine in Lebanon (June–December 2019). Structural equation modeling (SEM) was performed using SPSS AMOS v.24 to examine the structural relationship between each personality trait taken as independent variables, QOL as the dependent variable, and emotional intelligence as the mediator, among university students. The relative Chi-square (χ^2^/df), root mean square error of approximation (RMSEA) statistic, Tucker Lewis Index (TFI) and the comparative fit index (CFI) were used to evaluate the goodness-of-fit of the model.

**Results:**

Higher conscientiousness was significantly associated with more EI (Beta = 0.38; *p* < 0.001) and lower QOL (Beta = − 0.14; *p* = 0.025). The indirect relationships between conscientiousness, EI and QOL showed that EI mediated the association between conscientiousness and QOL (Beta = 0.17; 95% CI − 0.73 to − 0.004; *p* = 0.037). The fit indices of this model were adequate for χ^2^/df, RMSEA and CFI but not TLI. Higher openness to experience was significantly associated with more EI (Beta = 0.48; *p* < 0.001) and lower QOL (Beta = − 0.38; *p* < 0.001). The indirect relationships between openness to experience, EI and QOL showed that EI mediated the association between openness to experience and QOL (Beta = 0.30; 95% CI − 1.11 to − 0.03; *p* = 0.04). The fit indices were adequate for χ^2^/df and CFI but not RMSEA and TLI. EI did not mediate the association between the other three personality traits (extroversion, agreeableness and neuroticism) and QOL.

**Conclusion:**

This current research has shed considerable light on the nexus of associations between EI, personality traits and well-being, nonetheless, led to the creation of more puzzling questions. On the whole, it seems that EI and its components can be used as an evaluation instrument in relates with Lebanese medical students’ personality profile as a means of future training to improve quality of life during medical education.

## Background

Nowadays, medical schools and institutions are paying close attention to students' and employees’ quality of life (QOL) and personality traits due to various challenges in this dynamic environment. There is a great deal of research investigating how these two components in a person change over time, and if changes in one component (such personality) cause changes in the other (like QOL). However, few investigated what caused the participants' personalities and quality of life to change. To date, many organizations and businesses placed a very important criterion for selection of their employee. Emotional intelligence (EI) assessment is one of the tools used. As a respond to this issue, this research has been done and thus this paper is focusing on the link between QOL and personality traits, as well as a prospective mediational impact of EI.

### Importance of QOL

The stressful key characteristics of medical school can have an impact on medical students' physical and mental health, as well as their overall quality of life [1]. In fact, throughout their medical education, students are subject to academic pressure with the goal of obtaining a successful medical profession and coping with future uncertainty about medical practice and its related employment. Students may experience social, emotional, physical, and family issues, leading to negative repercussions on their academic performance and ability to study [2–6]. All these factors may contribute to lower quality of life among medical students [7]. It is, therefore, critical for medical schools and educators to understand students' QOL during their medical training [8]; research on QOL has the potential to enhance students' physical and mental health.

QOL is best defined as the overall well-being of individuals and societies, sketching out adverse and positive features of life [4]. QOL notices total life fulfillment when one's own life matches with an ideal standard level, including physical health, family, education, employment, wealth, religious beliefs, finance and the environment [3]. The importance of quality of life among medical students has been stressed since research has shown that it is related with an unhealthy lifestyle, mental health problems, academic failure, as well as having a detrimental influence on professional growth. Moreover, QOL is a multidimensional and relative concept influenced by time, place and individual values [4]. This highlights the significant interaction between physiological, behavioral, and emotional factors and several social and cultural factors such as religious beliefs, social networks involved in QOL, relative to each country. Actually, with regard to medical students, we were particularly concerned with the role of personality, and performance indicators such as academic results and clinical competence.

### Evidence for a personality—QOL relation

Personality is known as the blend of behavior, emotion, motivation, and thought patterns that characterize an individual [9]. Researchers have suggested five measurements of personality, which are extroversion, conscientiousness, agreeableness, neuroticism and openness to experience [10, 11]. The Big-Five Factor System has become a scientifically valuable paradigm in personality trait studies [12]. Extroversion refers to a person with high energy, sociable, with direct leadership roles, enthusiastic, assertive and with high measure of emotional expressiveness [13–15]. Conscientiousness is connected to a high degree of care, with goal-directed behavior, discipline, organization and precision [13–15]. Agreeableness is normally described by reliability, thoughtfulness, compassion and cooperativeness [13–15]. Neuroticism is regularly related to emotional insecurity, distress, moodiness, low adaptive capacity and bitterness [13–15]. Openness to experience is linked to innovativeness, wide scope of interest, inventive, impulsive and rational [13–15].

Personality traits, in reality, are features related to our motivational system's factory settings. In the absence of a significant impact from the surroundings, they determine what we are driven to accomplish [16]. Among the many possible variables that can be associated with QOL, researchers list personality traits. Indeed, a study of the research reveals that the relations between the Big Five personality traits and QOL are well established. It has been found that personality characteristics are good predictors of academic performance during medical school [17]. Furthermore, previous findings have denoted the vulnerability of certain personality traits to stress [18, 19] and poorer mental health [20]. Especially, medical students with pronounced levels of neuroticism were at expanded risk for depression [20] and suicidal ideation [21], suggesting that personality characteristics have a substantial impact on QOL.

### The potential process involved—Why is EI connected to personality traits and QOL?

A growing body of research has begun to investigate student academic state of achievement, specifically among medical students, and research‐based on useful or harmful agents’ approaches to narrowing performance gaps, has always been one of the researcher’s education system challenges. In addition to the relationship between personality and susceptibility to stress, which has been demonstrated to influence the QOL of medical students and has implications for performance, much needs to be discovered about how QOL is directly connected to other personality traits, and if these links are impacted indirectly by various personal or societal circumstances.

Researchers have revealed that the subjective experience and behavior can interfere with the emotional capacity of each individual [22]. Using the logic described earlier, our study's goal was to investigate a putative mediational mechanism involved in this connection. Individuals with high EI are mindful of their own feelings, ready to control their emotions well from overpowering pressure, depression, anxiety, or anger [23–26]. They are likewise eager to have a decent comprehension of others' feelings and utilize this capacity to oversee and change their behavior when communicating with others [27]. This ability model of EI posits four related skills: Self-Awareness, Self-Management, Social Awareness, and Relationship Management [28]. In brief, the four domains connect with the ability to know, differentiate and understand one’s own emotions; to control emotions for providing one’s own development; perceiving and understanding others' feelings; and managing relationships, i.e. dealing with the emotions of others [29]. In Goleman’s point of view, EI is the most important factor to determine the success of individuals, which means that people who develop qualities of human relationships are most likely to succeed in their daily life [30]. Emotional abilities and social skills under the title of EI exhibit a strong predictor of educational success. Therefore, EI as a predictor of success has become the central focus of medical education [31].

A vast amount of research has recorded higher academic achievement due to positive association between trait EI and medical education [32]. Various studies have reported that EI is associated with social network efficiency [33], positive communications [34], and better adaptation [35]. Furthermore, evidence shows that EI influences improvement in clinical practice factors, such as increased empathy in medical consultation, interactions between doctor and patient, clinical performance and patient satisfaction [36, 37]. These facts suggest that EI plays a crucial role in many areas relevant to the abilities of future medical practitioners.

Since it promotes well-being outcomes [38], optimism and hope [39], we hypothesized that EI may act as a possible mediator between Big Five Model personality traits and QOL. In the section that follows, we try to offer the main rationale for the critical assumption of the temporal precedence in the mediational scheme [40].

The correlation between personality traits and EI is the first significant link in the potential mediatory relationship. On a conceptual level, McCrae drew attention to links between EI and aspects of the Big Five Model's dimensions [41]. Furthermore, certain investigations have revealed that both variables are most likely intertwined [33, 42, 43]. EI has been found to have a negative and substantial relationship with neuroticism, as well as a positive and significant relationship with extraversion, openness, agreeableness, and conscientiousness [39, 44–47]. According to these studies, whereas neuroticism and extraversion were the main personality predictors of EI, agreeableness and openness were relatively weak. In turn, while performing a multiple regression, Petrides et al. discovered that all Big Five personality traits contribute considerably to the prediction of EI [48].

The second crucial element in the mediatory relationship is the link between EI and QOL. According to research, EI correlates with a higher feeling of personal well-being [49]. Several studies demonstrate that EI is a key predictor of life outcomes, therefore individuals with greater EI are better at handling work situations, building deeper connections with coworkers, and feeling more pleased and fulfilled in their life [50].

### The present study

Currently in the developed world, EI is increasingly made reference to in medicine. In Lebanon, this area is under research especially among the medical students in order to enhance a successful educational performance. The medical graduate is expected to be emotionally stable, empathetic to patients, good in counseling and having good leadership skills qualities [34]. Therefore, there is a critical need to identify factors associated with these special abilities and behaviors, since traditional tests used in medical admissions offer limited screening regarding these competences.

**Fig. 1:**
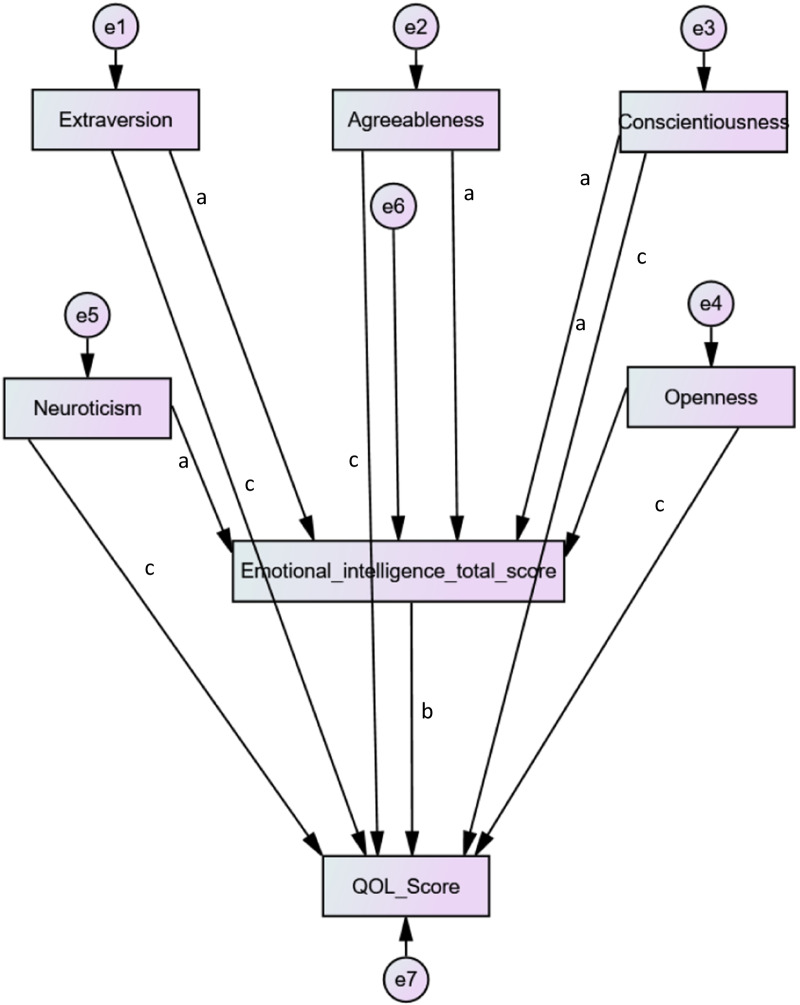
Quality of life, personality traits and emotional intelligence interconnections among medical students; **a** represents the coefficient between each personality trait and emotional intelligence; **b** the coefficient between emotional intelligence and quality of life (QOL); **c** the coefficient between each personality trait and QOL

Actually, medicine is a highly demanding career that appears to leave many medical students at the risk of stress, anxiety, depression and burnout [51, 52]. Hence, these entities can significantly affect the QOL among medical students. During the past couple of years, students in Lebanon endured multiple stressful events ranging from economic instability and unemployment [53], to lockdowns caused by the COVID-19 pandemic, in addition to the Beirut Port explosion [54]. Fares et al. rated stress among preclinical students at 62% and showed that 75% of Lebanese medical students suffered from burnout [55]. Obviously, getting into medical school has an effect on a student’s QOL because adaptation and lifestyle changes are needed. Taken together, specific Big-Five personality traits and EI facets may be both linked to medical student’s QOL [56]. In fact, researchers' theoretical insights and statistical findings have postulated that EI contributes to the establishment and maintenance of interpersonal relationships by utilizing the capacity to comprehend the feelings of others and convey to relate to them [57]. As a result, it can be presumed that personality domains (extraversion, openness, agreeableness, conscientiousness and neuroticism) can possibly lead to higher QOL when people experience satisfaction, have the ability to self-monitor in social situations, and understand the emotions and behaviors of others. However, to our knowledge, no studies have been done on the concurrent and prospective relationship between QOL-related personality traits and EI in Lebanese medical students. Accordingly, this study aimed to investigate the association between personality traits and QOL, taking into consideration the mediating role of EI, according to the theoretical framework in Fig. 1.

## Methods

### Participants

To reflect the target population, all participants were general medicine students aged 18 years and above, registered as full-time students in one of the 7 schools of medicine in Lebanon. The students were given a consent form stating the purpose of the study, benefits, risks and confidentiality of collected data. Participation in the study was voluntary, with no incentive. A pilot test was carried out on 15 students to check the clarity of the questionnaire. It is important to note that the 15 questionnaires related data were not entered in the final database.

The Epi info program (Centers for Disease Control and Prevention (CDC), Epi Info™) was exerted for the calculation of the minimal sample size entailed for our study, with an acceptable margin of error of 5%, a power of 80% and an expected variance of subjective well-being related to personality traits estimated by 14–28% [56] for 5531 general medicine student in Lebanon [58]. The results showed that a minimum of 293 participants is needed.

### Procedure

This research was conducted from June till December 2019 by using a descriptive cross-sectional design. The data were collected by completing an anonymous online or paper-based self-administered questionnaire upon the participant choice. The questionnaire was only available in English version since in their admission criteria; the 7 faculties of medicine in Lebanon, require a minimum level of good English knowledge. The same methodology has been used in a previous paper [59].

### Measures

The survey evaluated demographic information including age, gender, region, university, current year in medical education, academic performance, parental highest level of education, and personal medical history regarding physical and mental illnesses.

QOL was assessed using the 5-item *World Health Organization Well-Being Index* (WHO-5) [60], a short and generic global rating scale measuring subjective well-being. It is composed of 5 items, scored on a 6-point Likert scale (0 = at no time to 5 = all of the time). Higher scores reflect a better QOL. The Cronbach’s alpha value for this scale in this study was 0.925.

Personality traits were assessed using the *Big Five Personality Test*, regularly used in clinical psychology. Since its creation by John, Donahue, and Kentle (1991) [61], the five factor model was generally utilized in various nations including Lebanon; it portrays personality regarding the five-factor theory as per an individual’s reaction to 50 inquiries on a 5-point Likert scale: 1 (disagree) to 5 (agree). A score for each personality is determined to decide the major trait(s) in an individual character. The Cronbach’s alpha values were as follows: total scale (0.885), extroversion (0.880), conscientiousness (0.640), agreeableness (0.668), neuroticism (0.761) and openness to experience (0.718).

EI was evaluated utilizing the *Quick Emotional Intelligence Self-Assessment scale* [62]. The scale is divided into four domains: «emotional awareness, emotional management, social-emotional awareness, and relationship management». Every domain is made out of 10 inquiries, with answers measured on a 5-point Likert scale: 0 (never) to 4 (always). Higher scores indicate higher emotional intelligence (αCronbach = 0.950).

### Statistical analysis

All analyses were carried out using the Statistical Package for Social Sciences (version 24.0 with AMOS; IBM®, Armonk, NY, U.S.A.). No missing values were found in the database since all questions were required in the Google form. The normality of distribution of the QOL score was confirmed via a calculation of the skewness and kurtosis; values for asymmetry and kurtosis between − 1 and + 1 are considered acceptable in order to prove normal univariate distribution [63]. Structural equation modeling (SEM) was performed to examine the structural relationship between each personality trait taken as independent variables (X), QOL as the dependent variable (Y), and emotional intelligence as the mediator (M), among university students. The indirect effect of each personality trait on QOL through EI was deemed significant if the confidence interval did not pass by zero and if the fit indices of that model were adequate. The fit indices to evaluate the adequacy of the model were the following: the Relative Chi-square (χ^2^/df), root mean square error of approximation (RMSEA) statistic, Tucker Lewis Index (TFI) and the comparative fit index (CFI) were used to evaluate the goodness-of-fit of the model [32]. RMSEA values ≤ 0.06 or CFI and TFI values > 0.90 indicate a good-fitting model [32]. *p* < 0.05 was considered statistically significant.

## Results

### Sociodemographic and other characteristics of the participants

The overall average age of the medical students was 22.41 ± 2.20 years, with 130 (43.9%) males. The description of the scores was as follows: QOL (14.54 ± 6.31), EI (108.27 ± 24.90), extraversion (21.18 ± 8.96), agreeableness (28.01 ± 7.48), conscientiousness (25.20 ± 7.06), neuroticism (19.29 ± 8.94) and openness (27.36 ± 7.81). Other characteristics of the participants are summarized in Table 1.

**Table 1:** Sociodemographic and other characteristics of the participants (N = 296)

Variable	N (%)
*Gender*	
Male	130 (43.9%)
Female	166 (56.1%)
*Governorate*	
Beirut	28 (9.5%)
Mount Lebanon	124 (41.9%)
North	74 (25.0%)
South	37 (12.5%)
Bekaa	33 (11.1%)
*Monthly income*	
Low (< 1000 USD)	21 (7.1%)
Intermediate (1000–2000 USD)	133 (44.9%)
High (> 2000 USD)	142 (48.0%)

### Structural equation modeling

The fit indices of the five models taking each personality trait taken as independent variables, QOL as the dependent variable, and emotional intelligence as the mediator, are shown in Table 2. There was no multicollinearity between the variables entered in the model.

**Table 2:** Fit indices of the five models taking each personality trait taken as independent variables, QOL as the dependent variable, and emotional intelligence as the mediator, among university students

	χ^2^/df	TLI	CFI	RMSEA	90% CI
Extroversion	13.84/3 = 4.61	0.48	0.85	0.111	0.056–0.173
Agreeableness	16.08/3 = 5.36	0.49	0.55	0.122	0.068–0.183
Conscientiousness	7.14/3 = 2.38	0.78	0.94	0.068	0.000–0.135
Neuroticism	10.30/3 = 3.43	0.77	0.93	0.091	0.034–0.154
Openness to experience	14.45/3 = 4.82	0.71	0.91	0.114	0.06–0.175

**Fig. 2:**
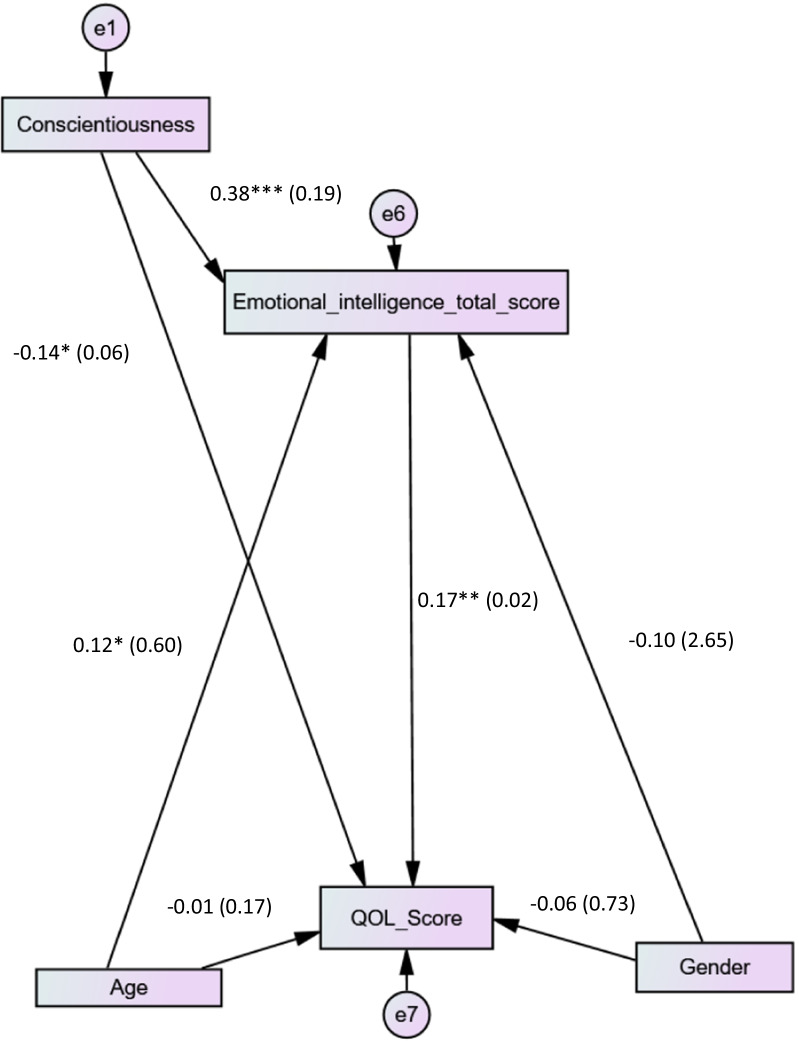
Conscientiousness, emotional intelligence and quality of life interconnections among medical students; **a** represents the coefficient between conscientiousness and emotional intelligence; **b** the coefficient between emotional intelligence and quality of life (QOL); **c** the coefficient between each conscientiousness and QOL. *p < 0.05; **p < 0.01; ***p < 0.001

Higher conscientiousness was significantly associated with more EI (Beta = 0.38; *p* < 0.001) and lower QOL (Beta = − 0.12; *p* = 0.025) (Fig. 2). The indirect relationships between conscientiousness, EI and QOL showed that EI mediated the association between conscientiousness and QOL (Beta = 0.17; 95% CI − 0.73 to − 0.004; *p* = 0.037). The fit indices of this model were adequate for χ^2^/df, RMSEA and CFI but not TLI.

**Fig. 3:**
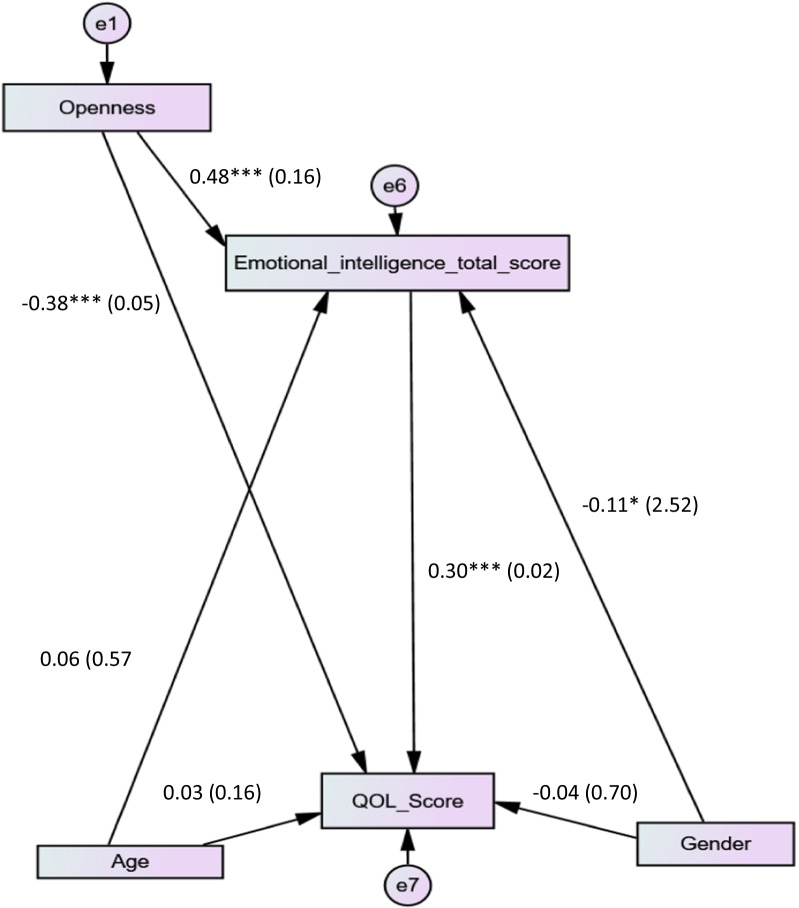
Openness to experience, emotional intelligence and quality of life interconnections among medical students; **a** represents the coefficient between openness to experience and emotional intelligence; **b** the coefficient between emotional intelligence and quality of life (QOL); **c** the coefficient between openness to experience and QOL. *p < 0.05; **p < 0.01; ***p < 0.001

Higher openness to experience was significantly associated with more EI (Beta = 0.48; *p* < 0.001) and lower QOL (Beta = − 0.38; *p* < 0.001) (Fig. 3). The indirect relationships between openness to experience, EI and QOL showed that EI mediated the association between openness to experience and QOL (Beta = 0.30; 95% CI − 1.11 to − 0.03; *p* = 0.04). The fit indices were adequate for χ^2^/df and CFI but not RMSEA and TLI.

Higher extroversion was significantly associated with more EI (Beta = 0.20; *p* < 0.001) and higher QOL (Beta = 0.36; *p* < 0.001). The indirect relationships between extroversion, EI and QOL showed that EI did not mediate the association between extroversion and QOL (Beta = 0.04; 95% CI − 0.33–0.05; *p* = 0.326).

Higher agreeableness was significantly associated with more EI (Beta = 0.12; *p* = 0.039) and lower QOL (Beta = − 0.12; *p* = 0.032). The indirect relationships between agreeableness, EI and QOL showed that EI did not mediate the association between agreeableness and QOL (Beta = 0.13; 95% CI − 0.69–0.02; *p* = 0.123).

Higher neuroticism was significantly associated with lower EI (Beta = − 0.17; *p* = 0.003) and higher QOL (Beta = 0.49; *p* < 0.001). The indirect relationships between neuroticism, EI and QOL showed that EI did not mediate the association between neuroticism and QOL (Beta = 0.20; 95% CI − 0.85–0.01; *p* = 0.057).

According to Mackinnon et al. [64], a mediating effect is deemed present if there were significant correlations of X to Y, X to M, M to Y (when X and M are in the same model) and if the regression coefficient of X to Y is higher in absolute value than the regression coefficient of X to Y in the presence of M. In the conscientiousness model, the first 3 rules were verified. Regarding the fourth one, the coefficients were − 0.14 and 0.06 for the direct and indirect associations respectively. In the openness to experience model, the same was verified for the first three rules. Regarding the fourth one, the coefficients were − 0.38 and 0.12 for the direct and indirect associations respectively. Therefore, the mediation assumptions for the 2 models were all verified.

To obtain the percentage of mediation, we divide the coefficient of the indirect by that of the direct association. Therefore, emotional intelligence mediated the association between conscientiousness and QOL by 42.86% and between openness to experience and QOL by 31.58%.

## Discussion

In our study, the relationships between QOL, EI and personality traits were assessed in a sample of general medicine students from different medical schools in Lebanon. This research permitted us to perceive medical students’ observations regarding their QOL during the medical program and analyze the mediating effect of EI between personality traits and QOL.

We initially found a slightly lack of well-being among Lebanese medical students (QOL total score = 14.54 ± 6.31). This finding is in line with a similar study that found high levels of psychological morbidity and depression among medical students [65]. In addition to the proven high prevalence of psychological distress, this study showed that the effects of distress can be detrimental to students’ well-being [65]. Medical school has long been recognized as involving various stressors and challenges that can affect students' well-being. In this sense, such finding might reflect dissatisfaction to the learning environment and/or the need for free time to study, take part in leisure pursuits, keep up relationships and gain enough rest [66].

Numerous studies showed that traits of extraversion and neuroticism strongly affect QOL score [56, 67, 68]. Generally, greater extraversion was related to better QOL, while greater neuroticism was connected to poorer QOL. Indeed, as Yik and Russell (2001) noted, neuroticism and extraversion are almost indistinguishable from two components of subjective well-being, negative and positive affect respectively [69]. However, in our study, contrary to the results of extraversion, which are consistent with previous studies, the expected negative association between neuroticism and QOL did not occur. These discrepancies between the results tend to suggest two alternative explanations; On the one hand, this contrast could be the product of our use of a different measurement tool for QOL and personality characteristics. On the other hand, findings of a cross-sectional study indicated that extraversion and neuroticism mediate the relationships between openness, conscientiousness, and agreeableness and affective well-being [70]. Thus, these traits do not exist in isolation. The positive association between neuroticism and QOL score found in our study could be explained by the possible occurrence of a combined or interaction effect.

Findings of this study revealed that openness to experience negatively affects the level of QOL. Interestingly, this result contradicts prior outcomes; previous results showed no consistent relationship between openness and subjective well-being [71], while others noticed that openness is positively associated with positive and negative affect, both of which are key elements of subjective well-being [72]. As stated earlier, the denoted negative association could be due to the idea that people with high openness prefer seeking a spectrum of life experiences, however, may feel overwhelmed if not satisfied from those experiences [73]. Indeed, pursuing a career in medicine is rigorous and demanding and may alter student’s yearning for exploring different life experiences.

Besides, higher EI was significantly associated with better QOL. Previous findings have proposed a hypothesis to explain the nexus between EI and subjective well-being. First, because EI give information about one’s relationship to the environment and others, deciphering and reacting to such information can direct action and thoughts in a manner that improves well-being [74]. Second, EI is associated with a lower affinity to encounter negative feelings and a higher inclination to encounter good feelings, adding more extravagant sense of subjective well-being [75].

The results of the mediation analysis showed that EI mediated the relationship of QOL with conscientiousness and openness to experience. These facts suggest that emotionally intelligent medical students who know how to handle their own and others’ emotions, and how to deal with emotions effectively, could cope with the demands and challenges of the medical program and, in turn, experience more academic satisfaction, thus achieve a better QOL. Actually, one of the major theories of personality postulates that personality traits are largely set by genetics, and, therefore, changes in personality traits can slow as other functions of maturation slow [76]. Consequently, a debate focusing on the extent to which subjective well-being could possibly improve through EI-Personality development, was entailed. Nelis et al. (2009) demonstrated that EI can be increased with training and that this gain in EI led to positive changes in students’ personality characteristics (increased agreeableness and extraversion and decreased neuroticism) compared to those not attending training. In fact, greater psychological well-being, prominent subjective health, better social relationships’ quality, and higher employability were reported among students enrolled in the training session [77]. The correlation between EI and a variety of positive outcomes among adolescents across the academic, social, psychological and career realms has been well documented [78]. These results support the idea that the intermediation role of EI in personality traits may be a consistent predictor of variance of general welfare.

In the medical context, many studies have shown that the personality traits are associated with various crucial areas which include approach to work, mental health, career success, learning approach and academic performance of medical students and professionals [79–82]. One key insight is that medical schools should pay closer attention to medical students who seem to have a history of poor academic achievement and may struggle to deal with the intensive environment of medical training. As a result, these students may require psychological assistance from the medical school from the beginning of their studies. This line of research could give rise to additional investigation of outcomes relating to academic performance resulting from emotional intelligence training.

### Clinical implications

The above stated results provides some preliminary contributions to research and practice. It indicates the need to evaluate counseling programs effects on QOL among medical students. Furthermore, it will be useful to explore whether these profiles remain constant during medical study or change due to academic environment and practice as part of a potential research agenda. A longitudinal study design would be ideal, to see the effect of training in the same students from first year to seventh year by comparing their own scores over years of study with a self-guided analysis that could assist students to know where they stand in the various areas of EI. Lastly, this study also represents a valuable contribution to the Lebanese medical society in order to implement variables such as EI and personality traits in the selection and recruitment process of health care professionals and students, implying better job satisfaction and performance.

### Limitations

This research has some drawbacks, considering these promising results. All variables were evaluated through a self-reported questionnaire and not through an ability-based measure, thus, the responses might lack precision and accuracy and may have been subject to reporting bias. Hence, in order to reduce this uncertainty, cross-ratings can be utilized in future studies. In addition, the survey was only available in English reflecting the great English knowledge required. As this study is cross-sectional, we can neither infirm causality nor confirm whether these associations would change over time; further this type of study reflects a lower internal validity. Selection bias could also venture on as in any voluntary study; with their already low academic performance, these non-participating medical students may be less motivated or discouraged. Finally, a residual confounding bias is also possible since not all factors associated with QOL were taken into consideration in this study.

### Conclusion

EI and personality traits showed a significant association with QOL of medical students. As stated above, according to most personality type theories, the individual's type is inborn and remains unchanged over the course of adult life [76, 83]. However, individuals can develop traits and habits that differ or even directly contradict the description of their type. These facts clearly pinpoint to the variable aspect of EI as an appealing criterion in the path between personality traits and quality of life and consequently determine the performance and academic abilities of medical students.

One way or the other, this current research has shed considerable light on the nexus of associations between EI, personality traits and quality of life, nonetheless, led to the creation of more puzzling questions. It seems that EI and its components can be used as an evaluation instrument in relates with Lebanese medical students’ personality profile as a means of future training to improve QOL during medical education. In fact, it would be compelling to look if this key concept could play a role regarding medical student's career/specialty choice and preferences, achievement motivation and academic success.

## Data Availability

All data generated or analyzed during this study are not publicly available to maintain the privacy of the individuals’ identities. The dataset supporting the conclusions is available upon request to the corresponding author.

## Abbreviations

EI: Emotional intelligence
QOL: Quality of life
CDC: Centers for Disease Control and Prevention
WHO: World Health Organization
SPSS: Statistical Package for Social Science
